# Gender Trends in Psychotropic Medication Use in Autism

**DOI:** 10.7759/cureus.26447

**Published:** 2022-06-30

**Authors:** Christopher R Huber, Zachary Fanaro, Varun Soti

**Affiliations:** 1 Medical School, Lake Erie College of Osteopathic Medicine, Elmira, USA; 2 Anatomy and Neuroanatomy, Lake Erie College of Osteopathic Medicine, Elmira, USA; 3 Pharmacology and Therapeutics, Lake Erie College of Osteopathic Medicine, Elmira, USA

**Keywords:** prescription medication, systematic literature review, sex differences, psychotropic drugs, autism spectrum disorders

## Abstract

Autism is a neurodevelopmental condition that includes differences in social communication and restrictive, repetitive behavior. Its diagnosis is far more common in men than women. Therefore, a female phenotype of autism might not concern caregivers or be detected early by clinical assessments. Given that medications address problematic behaviors rather than autism, different problems associated with autism necessitate other treatments. We reviewed existing literature on gender differences in psychotropic drug usage in autism patients and found that antidepressants, anticonvulsants, and mood stabilizers were more common in females, while stimulants and antipsychotics were predominant in males. This review highlights that autistic men and women receive different pharmacologic agents, likely attributable to gender-specific trends in presenting problematic behaviors.

## Introduction and background

Autism is a neurodevelopmental condition that includes differences in social communication and restrictive, repetitive behavior. According to the Centers for Disease Control and Prevention, one in 54 children has autism. Moreover, it is 4.3 times more prevalent in males than females [1]. Although the complete explanation for the male predominance is unclear, the difference may be partly due to innate physiologic sex differences. A complementary reason for the male predominance is that autistic women present differently than autistic men. Autistic women are more likely to have an intellectual disability (intelligence quotient (IQ) ≤70) compared to their male counterparts, 39% versus 32%, respectively [1].

Interestingly, women with average IQ and without language delays may remain unrecognized due to milder displays of social and communication difficulties [2]. This lack of clinical assessment of autism in females could be due to social camouflaging. It refers to the idea that autistic individuals (especially females) can better conceal their social difficulties than their male counterparts, leading to fewer apparent symptoms and a later diagnosis (if diagnosed at all) [3].

The gender difference in autism expression not only leads to a diagnosis with autism spectrum disorder at different life stages in male and female patients but can initiate different trends in medication usage. Because no specific drugs target the core symptoms of autism, including restrictive, repetitive behavior and social communication, prescription patterns are based on the problem behaviors. Therefore, several classes of drugs are prescribed to autistic patients, including antidepressants, stimulants, attention-deficit hyperactivity disorder (ADHD) medications, and mood stabilizers [4].

Different antidepressants used to treat autism include selective serotonin reuptake inhibitors (SSRIs), selective norepinephrine reuptake inhibitors, tricyclic antidepressants, monoamine oxidase inhibitors, and other antidepressants. SSRIs are used for anxiety and depression comorbidities and repetitive behavior or rigidity in autistic patients. Moreover, stimulants and other ADHD medications treat hyperactivity, distractibility, and impulsivity in autistic patients, such as atomoxetine and alpha-2-adrenergic agonists (for example, mirtazapine, clonidine, and guanfacine). Mood stabilizers are classified as either anticonvulsant or non-anticonvulsant. They have utility in treating inattention and hyperactivity and maladaptive and severe problems or disruptive behaviors associated with autism. Divalproex sodium, a combination of valproate and valproic acid, has been extensively used. Other agents, such as lamotrigine, levetiracetam, oxcarbazepine, and topiramate, are ideal for subsets of autistic populations, chiefly those with comorbid seizure disorders, epilepsy, and who have intolerable adverse effects to atypical antipsychotics. The non-anticonvulsant mood stabilizers include lithium and the three generations of antipsychotics. Lithium is helpful in a minority of autistic patients for mood lability [5].

The complexities in the challenging behaviors exhibited by autistic males and females contribute to prescribing a wide range of psychotropic medications. Understanding these prescription patterns can advance our understanding of these different patterns of autism and lead to better treatment and symptom management. Therefore, this review aimed to identify the gender-specific trends in psychotropic medication usage in autism management. We primarily focused on evaluating whether autistic men and women are recipients of different psychotropic medications and whether this gender difference in using specific categories of medicines sheds light on the female phenotype of autism.

## Review

Literature search and study selection

**Figure 1:**
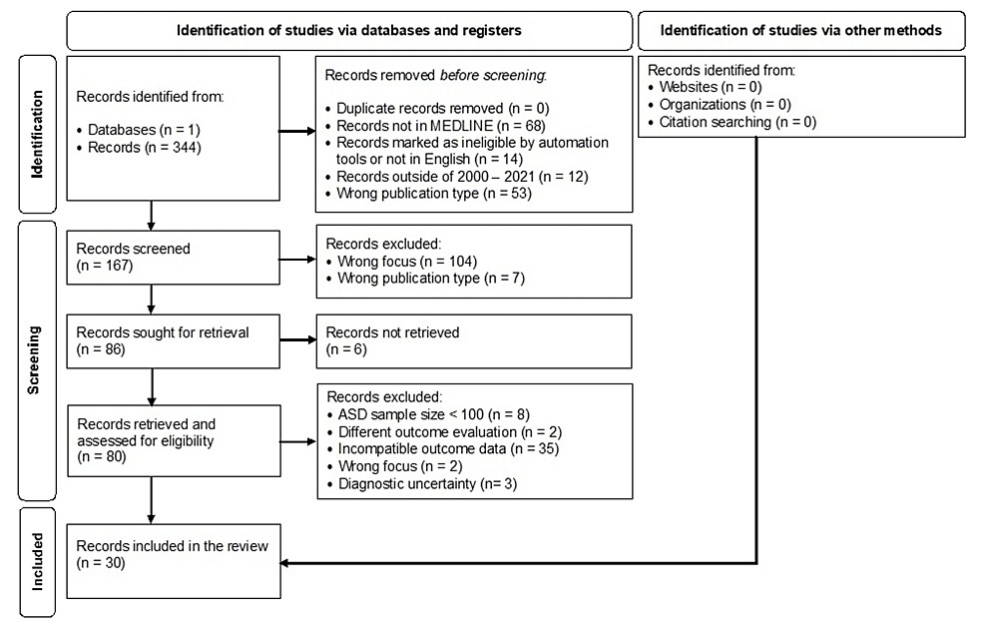
Literature search and study selection. This review utilized PubMed and followed PRISMA guidelines to search for clinical studies on the use of psychotropic substances in autistic patients. The search strategy involved using specific search terms in different combinations. The keywords were limited to “Autism,” “Prescription Use,” “Prescription Trend,” “Prescription Pattern,” “Prescription Management,” “Medication Use,” “Medication Trend,” “Medication Pattern,” “Medication Management,” “Pharmacologic Use,” “Pharmacologic Management,” “Psychotropic Use,” “Psychotropic Management,” “Pharmacy Use,” “Pharmacy Management,” “Psychiatric Use,” “Psychiatric Management,” “Prescribing Pattern,” “Prescribing Trend,” “Psychotropic Medication,” “Psychotropic Drug,” “Prescription Rate,” and “Drug Use.” By using filters and inclusion criteria, including articles written in English, and complete clinical studies focused on psychotropic medications in autistic males and females, the number of studies was narrowed down to 30. ASD: autism spectrum disorder; MEDLINE: Medical Literature Analysis and Retrieval System Online; PRISMA: Preferred Reporting Items for Systematic Reviews and Meta-Analyses

We conducted a literature search from July 2021 through January 2022, following the evidence-based guidelines for systematic reviews described in the Preferred Reporting Items for Systematic reviews and Meta-Analyses (PRISMA) [6]. We used PubMed and a PRISMA checklist. As illustrated in Figure 1, the search strategy included articles written in English and published between 2000 and 2021 and excluded systematic reviews, meta-analyses, case reports, and editorials. Search terms included “autism” AND “prescription use” OR “prescription trend” OR “prescription pattern” OR “prescription management” OR “medication use” OR “medication trend” OR “medication pattern” OR “medication management” OR “pharmacologic use” OR “pharmacologic management” OR “psychotropic use” OR “psychotropic management” OR “pharmacy use” OR “pharmacy management” OR “psychiatric use” OR “psychiatric management” OR “prescribing pattern” OR “prescribing trend” OR “psychotropic medication” OR “psychotropic drug” OR “prescription rate” OR “drug use.” Of the 167 studies screened, we excluded 111 and retrieved 80. We did not include studies examining complementary or alternative health medicine because this review focused only on psychotropic medications. Of the 80 articles retrieved, 30 met the inclusion criteria.

Use of psychotropic medications

Of the 30 studies reviewed, 25 assessed the male versus female usage of any psychotropic medication (Table 1). Of these 25 studies, six reported only crude percentages and did not assess statistical significance. In the other 19 studies that analyzed the statistical significance, 13 did not perform statistical analysis, while six showed a difference between psychotropic medication use between male and female autistic patients. Four of the six studies found that males were significantly more likely to take any psychotropic medication, while two found that females were significantly more likely to take any psychotropic medication.

**Table 1:** Use of psychotropic drugs male versus female. Significant results are highlighted in bold. Some studies did not report specific data points, though results were deemed substantial or not substantial. Other studies reported crude percentages and did not perform statistical analyses. Their results are neither in bold nor stated as ns. ^a^ Odds ratio = 1.71, 95% confidence interval = 1.47–2.01; ^b^ χ2 = 110.86, p < 0.001; ^c^ Odds ratio = 0.74, 95% confidence interval = 0.66–0.83, p < 0.01; ^d^ p < 0.0001; ^e^ Odds ratio = 0.94, 95% confidence interval = 0.90–0.98, p = 0.0093; ^f^ p = 0.004; ^g^ No significant variation in medication use by gender. ADI-R: Autism Diagnostic Interview-Revised; ADOS-2: Autism Diagnostic Observation Schedule-Second Edition; ASD: autism spectrum disorder; ATN: Autism Treatment Network; CPRD: Clinical Practice Research Datalink; DSM-V: Diagnostic and Statistical Manual of Mental Disorders-Fifth Edition; DSM-IV-TR: Diagnostic and Statistical Manual of Mental Disorders-Fourth Edition-Text Revision; F: female; FFS: fee-for-service; ICD-9: International Statistical Classification of Diseases and Related Health Problems-Ninth Revision; ICD-10-GM: International Statistical Classification of Diseases and Related Health Problems-Tenth Revision-German Modification; M: male; ns: not significant; NS-CSHCN: National Survey of Children with Special Health Care Needs; RAMQ: Régie de l’assurance maladie du Québec; THIN: The Health Improvement Network

Author(s) (Year)	Sample size	Location	Means for participant identification (Year)	Diagnosis	Age (Year)	Crude % (M vs. F)	Significance
Croteau et al. (2017) [7]	2,989	Canada	﻿RAMQ (1998–2010)	ICD-9 code 299.X (excluding 299.2)	1–25	Not reported	ns
Croteau et al. (2019) [8]	1,227	Canada	﻿RAMQ (1998–2010)	ICD-9 code 299.X	1–25	Not reported	ns
Dalsgaard et al. (2014) [9]	9,698	Denmark	Danish Civil Registration System (1990–2001)	ICD-10 codes, F84.0, F84.1, F84.8, or F84.9	4–20	Not reported	M > F (ref. F)^a^
Bachmann et al. (2013) [10]	1,124	Germany	﻿Gmünder ErsatzKasse (2009)	ICD-10-GM F84.0, F84.1, F84.5, F84.8, F84.9	0–24	33% vs. 33%	Not reported
Memari et al. (2012) [11]	345	Iran	Autism-specific schools in Tehran, Iran (2010–2011)	DSM-IV-TR criteria and ADI-R	7–14	80% vs. 78%	ns
McConkey et al. (2021) [12]	1,133	Iran	Survey of Tehran Province and the City of Tehran (2005–2019)	DSM-V criteria and ADI-R	2–17	77% vs. 65%	Not reported
Meiri et al. (2018) [13]	211	Israel	Soroka University Medical Center	DSM-IV and DSM-V Criteria	1–6	Not reported	ns
Fusar-Poli et al. (2019) [14]	195	Italy	Two outpatient clinics dedicated to adolescents and adults with ASD (2018)	DSM-V criteria and ADOS-2 and/or ADI-R	14–58	62% vs. 47%	ns
Hong et al. (2017) [15]	17,606	Korea	Korean National Health Insurance Claims Database (2009–2013)	ICD-10, F84.0, F84.1, F84.2, F84.3, F84.4, F84.5, F84.8, F84.9	0–18	32% vs. 24%	M > F^b^
Houghton et al. (2018) [16]	10,856	UK	Clinical Practice Research Datalink (2015)	ASD diagnoses recorded in CPRD	3+	Not reported	M < F (ref. F)^c^
Alfageh et al. (2020) [17]	20,194	UK	THIN Database	ASD diagnoses recorded in THIN	All	31% vs. 37%	M < F^d^
Murray et al. (2014) [18]	5,651	UK	THIN Database (1992–2008)	ASD diagnoses recorded in THIN	0–24	28% vs. 32%	Not reported
Mayes et al. (2020) [19]	1,407	USA	Psychiatry Diagnostic Clinic	DSM-IV or DSM-V	2–17	35% vs. 29%	Not reported
Spencer et al. (2013) [20]	33,565	USA	Administrative Claims Database (2001–2009)	ICD-9-CM codes 299.0x, 299.8x, and 299.9x	0–20	64% vs. 64%	ns
Wink et al. (2018) [21]	350	USA	Autism Inpatient Collection	ADOS-2	4–21	Not reported	ns
Mandell et al. (2008) [22]	60,641	USA	Centers for Medicare and Medicaid Services Medicaid Analytic Extract (2001)	ICD-9 code 299.00, 299.8, or 299.9 associated with Medicaid reimbursed claim in 2001	0–21	56% vs. 55%	M > F^e^
Khanna et al. (2013) [23]	1,330	USA	Medicaid FFS Administrative-Claims	ICD-9 Autism in records	0–65	66% vs. 66%	Not reported
Kamimura-Nishimura et al. (2017) [24]	1,083	USA	National Ambulatory Medical Care Surveys (1994–2009)	ICD-9	2–18	54% vs. 49%	ns
Esler et al. (2019) [25]	1,265	USA	National Core Indicators Program (2012–2013)	ASD in records	18–96	Not reported	M > F^f^
Frazier et al. (2011) [26]	890	USA	National Longitudinal Transition Study-2 (2000)	Based on special education assignment	13–17	Not reported	ns
Madden et al. (2017) [27]	7,901	USA	Subset of the Health Care Systems Research Network (2009–2010)	ICD-9 code 299.0, 299.8, or 299.9	1–17	Not reported	ns
Zuckerman et al. (2015) [28]	1,420	USA	Pathways Survey (2011) and NS-CSHCN (2009-2010)	Parent reported	6–17	53% vs. 51%	Not reported
Houghton et al. (2017) [29]	93,639	USA	Truven Health MarketScan®﻿﻿ Commercial and Multi-State Medicaid Database (2014)	ICD-9 codes 299.0x, 299.8x, 299.9x	3+	Not reported	ns
Coury et al. (2012) [30]	2,853	USA and Canada	Autism Speaks ATN (2007-2011)	DSM-IV-TR criteria and ADOS	2–17	Not reported	ns^g^
Ziskind et al. (2020) [31]	613	USA and Canada	Not reported	Autism Speaks ATN	3–6	17% vs. 15%	ns

Use of antidepressants

Of the 30 studies included, nine reported antidepressant use in males and females (Table 2). Two studies reporting only a crude percentage found a greater likelihood of females receiving antidepressants, although statistical significance was not assessed. Five separate studies found no significant difference. Two studies detailed that females used significantly more antidepressants than males.

**Table 2:** Use of antidepressants male versus female. Significant results are highlighted in bold. ^a^ χ2 = 97.92; p < 0.001; ^b^ p < 0.05; ^c ^Specific statistics not reported but noted as females more likely; ^d^ No significant variation in medication use by gender. Note that only selective serotonin reuptake inhibitors were assessed. ADI-R: Autism Diagnostic Interview-Revised; ADOS: Autism Diagnostic Observation Schedule; ADOS-2: Autism Diagnostic Observation Schedule-Second Edition; ASD: autism spectrum disorder; ATN: Autism Treatment Network; DSM-IV-TR: Diagnostic and Statistical Manual of Mental Disorders-Fourth Edition-Text Revision; F: female; FFS: fee-for-service; ICD-9: International Statistical Classification of Diseases and Related Health Problems-Ninth Revision; ICD-10: International Statistical Classification of Diseases and Related Health Problems-Tenth Revision; M: male; ns: not significant; THIN: The Health Improvement Network

Author(s) (Year)	Sample size	Location	Means for participant identification (Year)	Diagnosis	Age (Year)	Crude % (M vs. F)	Significance
Memari et al. (2012) [11]	345	Iran	Autism-specific schools in Tehran, Iran (2010–2011)	DSM-IV-TR criteria and ADIR	7–14	8.7% vs. 8.7%	ns
Hong et al. (2017) [15]	17,606	Korea	Korean National Health Insurance Claims Database (2009–2013)	ICD-10, F84.0, F84.1, F84.2, F84.3, F84.4, F84.5, F84.8, F84.9	0–18	Not reported	M < F^a^
Alfageh et al. (2020) [17]	20,194	UK	THIN Database	ASD diagnoses recorded in THIN	All	7.6% vs. 14.5%	Not reported
Wink et al. (2018) [21]	350	USA	Autism Inpatient Collection	ADOS-2	4–21	Not reported	ns
Mandell et al. (2008) [22]	60,641	USA	Centers for Medicare and Medicaid Services Medicaid Analytic Extract (2001)	ICD-9 code 299.00, 299.8, or 299.9	0–21	25 vs. 25%	ns
Stein et al. (2012) [32]	27,421	USA	Medicaid Claims Data (2006–2010)	ICD-9 codes 299.0–299.8	0–17	Not reported	ns
Khanna et al. (2013) [23]	1,330	USA	Medicaid FFS Administrative-Claims	ICD-9 Autism in records	0–65	18.3% vs. 24.2%	M < F^b^
Madden et al. (2017) [27]	7,901	USA	Subset of the Health Care Systems Research Network (2009–2010)	ICD-9 code 299.0, 299.8, or 299.9	1–17	32.3% vs. 36.5%	Not reported^c^
Coury et al. (2012) [30]	2,853	USA and Canada	Autism Speaks ATN (2007–2011)	DSM-IV-TR criteria and ADOS	2–17	Not reported	ns^d^

Use of stimulants and other ADHD drugs

Of the 30 clinical studies analyzed, 13 estimated the use of stimulants and other ADHD drug prescriptions (Table 3). Two studies reported only crude percentages and did not perform statistical analysis, though both demonstrated a more significant share of males who received ADHD medications than females. Three studies found no statistical difference between usage. Eight studies found that males were prescribed considerably more stimulants or other ADHD drugs than females.

**Table 3:** Use of stimulants and other ADHD drugs male versus female. Significant results are highlighted in bold. ^a^ p < 0.0001; ^b^ Odds ratio = 1.71 (95% confidence interval = 1.47–2.01); ^c^ Noted significant, but statistics not reported; ^d^ p = 0.021; ^e^ p ≤ 0.02; ^f^ χ2 = 97.92, p < 0.001; ^g^ β = - 0.16, p = 0.011; ^h^ p < 0.05; ^i^ Specific statistics not reported but noted as males more likely; ^j^ No significant variation in medication uses by gender. ADHD: attention-deficit hyperactivity disorder; ADI-R: Autism Diagnostic Interview-Revised; ADOS: Autism Diagnostic Observation Schedule; ADOS-2: Autism Diagnostic Observation Schedule-Second Edition; ASD: autism spectrum disorder; ATN: Autism Treatment Network; DSM-IV-TR: Diagnostic and Statistical Manual of Mental Disorders-Fourth Edition-Text Revision; F: female; FFS: fee-for-service; ICD-9: International Statistical Classification of Diseases and Related Health Problems-Ninth Revision; ICD-10: International Statistical Classification of Diseases and Related Health Problems-Tenth Revision; ICD-10-GM: International Statistical Classification of Diseases and Related Health Problems-Tenth Revision-German Modification; M: male; ns: not significant; RAMQ: Régie de l’assurance maladie du Québec; THIN: The Health Improvement Network

Author(s) (Year)	Sample size	Location	Means for participant identification (Year)	Diagnosis	Age (Year)	Crude % (M vs. F)	Significance
Croteau et al. (2017) [7]	2,989	Canada	﻿RAMQ (1998–2010)	ICD-9 code 299.X (excluding 299.2)	1–25	Not reported	M > F^a^
Dalsgaard et al. (2014) [9]	9,698	Denmark	Danish Civil Registration System (1990–2001)	ICD-10 codes F84.0, F84.1, F84.8, or F84.9	4–20	Not reported	M > F (ref. F)^b^
Bachmann et al. (2013) [10]	1,124	Germany	﻿Gmünder ErsatzKasse (2009)	ICD-10-GM (German Modification) F84.0, F84.1, F84.5, F84.8, F84.9	0–24	14.2% vs. 7.2	M > F^c^
Memari et al. (2012) [11]	345	Iran	Autism-specific schools in Tehran, Iran (2010–2011)	DSM-IV-TR criteria and ADI-R	7–14	17.4% vs. 0%	M > F^d^
Satoh et al. (2016) [33]	3,276	Japan	Japan Medical Data Center	ICD-10, F84.0, F84.1, F84.2, F84.3, F84.4, F84.5, F84.8, F84.9	2–18	Not reported	M > F^e^
Hong et al. (2017) [15]	17,606	Korea	Korean National Health Insurance Claims Database (2009–2013)	ICD-10, F84.0, F84.1, F84.2, F84.3, F84.4, F84.5, F84.8, F84.9	0–18	Not reported	M > F^f^
Alfageh et al. (2020) [17]	20,194	UK	THIN Database	ASD diagnoses recorded in THIN	All	6.4% vs. 3.5%	Not reported
Wink et al. (2018) [21]	350	USA	Autism Inpatient Collection	ADOS-2	4–21	Not reported	ns
Mandell et al. (2008) [22]	60,641	USA	Centers for Medicare and Medicaid Services Medicaid Analytic Extract (2001)	ICD-9 code 299.00, 299.8, or 299.9 associated with Medicaid reimbursed claim in 2001	0–21	24% vs. 17%	ns
Stein et al. (2012) [32]	27,421	USA	Medicaid claims data (2006–2010)	ICD-9 codes 299.0–299.8 from July 1, 2006 to June 30, 2010	0–17	Not reported	M > F^g^
Khanna et al. (2013) [23]	1,330	USA	Medicaid FFS Administrative-Claims	ICD-9 Autism in records	0–65	33.3% vs. 25.3%	M > F^h^
Madden et al. (2017) [27]	2,979	USA	Subset of the Health Care Systems Research Network (2009–2010)	ICD-9 code 299.0, 299.8, or 299.9	12–17	44.3% vs. 30.5%	Not reported^i^
Coury et al. (2012) [30]	2,853	USA and Canada	Autism Speaks ATN (2007–2011)	DSM-IV-TR criteria and ADOS	2–17	Not reported	ns^j^

Use of antipsychotics (neuroleptics)

Of the 30 studies reviewed, 10 reported usage of antipsychotics (neuroleptics) (Table 4). Three of the 10 studies determined crude percentages and did not report statistical significance. Five studies found no statistically significant impact of gender on antipsychotic usage. However, two studies reported that males received significantly more neuroleptics than females.

**Table 4:** Use of antipsychotics (neuroleptics) male versus female. Significant results are highlighted in bold. ^a ^p = 0.003; ^b^ χ2 = 97.92, p < 0.001; ^c^ No significant variation in medication uses by gender; ^d^ Gender was not associated with atypical antipsychotic prescription (second-generation antipsychotics). Other antipsychotics were not reported. ADI-R: Autism Diagnostic Interview-Revised; ADOS: Autism Diagnostic Observation Schedule; ADOS-2: Autism Diagnostic Observation Schedule-Second Edition; ASD: autism spectrum disorder; ATN: Autism Treatment Network; DSM-IV: Diagnostic and Statistical Manual of Mental Disorders-Fourth Edition; DSM-IV-TR: Diagnostic and Statistical Manual of Mental Disorders-Fourth Edition-Text Revision; F: female; FFS: fee-for-service; ICD-9: International Statistical Classification of Diseases and Related Health Problems-Ninth Revision; ICD-10: International Statistical Classification of Diseases and Related Health Problems-Tenth Revision; M: male; ns: not significant; THIN: The Health Improvement Network

Author(s) (Year)	Sample size	Location	Means for participant identification (Year)	Diagnosis	Age (Year)	Crude % (M vs. F)	Significance
Memari et al. (2012) [11]	345	Iran	Autism-specific schools in Tehran, Iran (2010–2011)	DSM-IV-TR criteria and ADI-R	7–14	64.1% vs. 30.4%	M > F^a^
Hong et al. (2017) [15]	17,606	Korea	Korean National Health Insurance Claims Database (2009–2013)	ICD-10, F84.0, F84.1, F84.2, F84.3, F84.4, F84.5, F84.8, F84.9	0–18	Not reported	M > F^b^
Alfageh et al. (2020) [17]	20,194	UK	THIN Database	ASD diagnoses recorded in THIN	All	3.9% vs. 4.7%	Not reported
Wink et al. (2018) [21]	350	USA	Autism Inpatient Collection	ADOS-2	4–21	Not reported	ns
Mandell et al. (2008) [22]	60,641	USA	Centers for Medicare and Medicaid Services Medicaid Analytic Extract (2001)	ICD-9 code 299.00, 299.8, or 299.9 associated with Medicaid reimbursed claim in 2001	0–21	32% vs. 28%	ns
Stein et al. (2012) [32]	27,421	USA	Medicaid claims data (2006–2010)	ICD-9 codes 299.0–299.8 from July 1, 2006 to June 30, 2010	0–17	Not reported	ns
Khanna et al. (2013) [23]	1,330	USA	Medicaid FFS Administrative-Claims	ICD-9 Autism in records	0–65	39.75% vs. 39.5%	Not reported
Madden et al. (2017) [27]	2,979	USA	Subset of the Health Care Systems Research Network (2009–2010)	ICD-9 code 299.0, 299.8, or 299.9	12–17	33.1 vs. 32.1%	Not reported
Coury et al. (2012) [30]	2,853	USA and Canada	Autism Speaks Autism Treatment Network (ATN) (2007–2011)	DSM-IV-TR criteria and ADOS	2–17	Not reported	ns^c^
Lake et al. (2017) [34]	4,749 and 401	USA and Canada	ATN Physician Reported	DSM-IV	2–11 and 12–17	5.5% vs. 5.1% and 18.3% vs. 14.5%	ns^d^ and ns

Use of mood stabilizers or anticonvulsants

Of the 30 clinical studies, 10 investigated mood stabilizers or anticonvulsant use (Table 5). Two studies presented only a raw percentage of medication usage. Both found that females were more likely to use anticonvulsants or mood stabilizers, although investigators did not perform statistical analysis. In addition, Croteau et al. (2017) [7] reported that gender impacted the use of anticonvulsants but did not specify the nature of the relationship. However, three studies showed no significant impact of gender on mood stabilizer usage. On the contrary, four studies found that females were significantly more likely to use mood stabilizers or anticonvulsants than males.

**Table 5:** Use of mood stabilizers or anticonvulsants male versus female. Significant results are highlighted in bold. ^a^ Specific statistics not reported but noted that the gender impacted the anticonvulsant use; ^b^ Noted significant, but statistics not reported. It reported data for anticonvulsants; ^c^ p = 0.014. It reported mood stabilizers/anticonvulsants as one category; ^d^ p ﻿≤﻿ 0.02. It reported data for anticonvulsants; ^e^ χ2 = 97.92; p < 0.001. It reported data for mood stabilizers; ^f^ The study reported data for anticonvulsants; ^g^ The study reported data for mood stabilizers; ^h^ The study reported data for mood stabilizers;^ i ^The study reported data for mood stabilizers; ^j ^Specific statistics were not reported, but noted as females more likely; ^k^ Specific statistics were not reported but noted as females more likely. ADI-R: Autism Diagnostic Interview-Revised; ADOS: Autism Diagnostic Observation Schedule; ADOS-2: Autism Diagnostic Observation Schedule-Second Edition; ASD: autism spectrum disorder; F: female; ICD-9: International Statistical Classification of Diseases and Related Health Problems-Ninth Revision; ICD-10: International Statistical Classification of Diseases and Related Health Problems-Tenth Revision; ICD-10-GM: International Statistical Classification of Diseases and Related Health Problems-Tenth Revision-German Modification; M: male; ns: not significant; RAMQ: Régie de l’assurance maladie du Québec; THIN: The Health Improvement Network

Author(s) (Year)	Sample size	Location	Means for participant identification (Year)	Diagnosis	Age (Year)	Crude % (M vs. F)	Significance
Croteau et al. (2017) [7]	2,989	Canada	﻿RAMQ (1998–2010)	ICD-9 code 299.X (excluding 299.2)	1–25	Not reported	Yes^a^
Bachmann et al. (2013) [10]	1,124	Germany	﻿Gmünder ErsatzKasse (2009)	ICD-10-GM (German Modification) F84.0, F84.1, F84.5, F84.8, F84.9	0–24	7.7% vs. 13.4%	M < F^b^
Memari et al. (2012) [11]	345	Iran	Autism-specific schools in Tehran, Iran (2010–2011)	DSM-IV-TR criteria and ADI-R	7–14	29.3% vs. 56.5%	M < F^c^
Satoh et al. (2016) [33]	3,276	Japan	Korean National Health Insurance Claims Database (2009–2013)	ICD-10, F84.0, F84.1, F84.2, F84.3, F84.4, F84.5, F84.8, F84.9	2–18	Not reported	M < F^d^
Hong et al. (2017) [15]	17,606	Korea	Korean National Health Insurance Claims Database (2009–2013)	ICD-10, F84.0, F84.1, F84.2, F84.3, F84.4, F84.5, F84.8, F84.9	0–18	Not reported	M < F^e^
Alfageh et al. (2020) [17]	20,194	UK	THIN Database	ASD diagnoses in THIN	All	2.6% vs. 3.9%	Not reported^f^
Wink et al. (2018) [21]	350	USA	Autism Inpatient Collection	ADOS-2	4–21	Not reported	ns^g^
Mandell et al. (2008) [22]	60,641	USA	Centers for Medicare and Medicaid Services Medicaid Analytic Extract (2001)	ICD-9 code 299.00, 299.8, or 299.9 associated with Medicaid reimbursed claim in 2001	0–21	20% vs. 24%	ns^h^
Stein et al. (2012) [32]	27,421	USA	Medicaid claims data (2006–2010)	ICD-9 codes 299.0–299.8 from July 1, 2006 to June 30, 2010	0–17	Not reported	ns^i^
Madden et al. (2017) [27]	7,901	USA	Subset of the Health Care Systems Research Network (2009–2010)	ICD-9 code 299.0, 299.8, or 299.9	5–11; 12–17	5.5% vs. 9.2%; 14.0 vs. 20.0%	Not reported^j^; Not reported^k^

Use of anxiolytics, hypnotics, and sedatives

A total of eight studies examined the use of anxiolytics, hypnotics, and sedatives (Table 6). Alfageh et al. (2020) [17] and Mandell et al. (2008) [22] reported only crude statistics. Two studies found no significant difference, while four studies showed a substantial impact of gender on the prescription patterns of these drugs. Croteau et al. (2017) [7] demonstrated that gender impacted the use of anxiolytics but did not specify the nature of the relationship. The remaining three studies found that females used significantly more anxiolytics, hypnotics, and/or sedatives than males.

**Table 6:** Use of anxiolytics, hypnotics, and sedatives male versus female. Significant results are highlighted in bold. ^a^ The category was anxiolytics. Although the direction was not specific, gender impacted the use of anxiolytics; ^b^ Statistics were not reported but noted significant (specific for anxiolytics). The category was anxiolytics; ^c^ p = 0.025. Study category was sedatives/hypnotics; ^d^ χ2 = 97.92; p < 0.001. The category was anxiolytics; ^e^ It reported data on anxiolytics and hypnotics; ^f^ Category was anxiolytics; ^g^ Category was hypnotics; ^h^ Study category was anxiolytics; ^i ^It addressed data on anxiolytics and sedatives;^ j ^The category was anxiolytics; ^k^ Category was sedatives; ^l ^Category was anxiolytics/hypnotics/sedatives. ADI-R: Autism Diagnostic Interview-Revised; ADOS-2: Autism Diagnostic Observation Schedule-Second Edition; ASD: autism spectrum disorder; F: Female; FFS: fee-for-service; ICD-9: International Statistical Classification of Diseases and Related Health Problems-Ninth Revision; ICD-10: International Statistical Classification of Diseases and Related Health Problems-Tenth Revision; ICD-10-GM: International Statistical Classification of Diseases and Related Health Problems-Tenth Revision-German Modification; M: male; ns: not significant; RAMQ: Régie de l’assurance maladie du Québec; THIN: The Health Improvement Network

Author(s) (Year)	Sample size	Location	Means for participant identification (Year)	Diagnosis	Age (year)	Crude % (M vs. F)	Significance
Croteau et al. (2017) [7]	2,989	Canada	﻿RAMQ (1998–2010)	ICD-9 code 299.X (excluding 299.2)	1–25	Not reported	Yes^a^
Bachmann et al. (2013) [10]	1,124	Germany	﻿Gmünder ErsatzKasse (2009)	ICD-10-GM (German Modification) F84.0, F84.1, F84.5, F84.8, F84.9	0–24	5.8% vs. 9.8%	M < F^b^
Memari et al. (2012) [11]	345	Iran	Autism-specific schools in Tehran, Iran (2010–2011)	DSM-IV-TR criteria and ADI-R	7–14	1.1% vs. 13%	M < F^c^
Hong et al. (2017) [15]	17,606	Korea	Korean National Health Insurance Claims Database (2009–2013)	ICD-10, F84.0, F84.1, F84.2, F84.3, F84.4, F84.5, F84.8, F84.9	0–18	Not reported	M < F^d^
Alfageh et al. (2020)^e ^[17]	20,194	UK	THIN Database	ASD diagnoses recorded in THIN	All	1.0% vs. 1.8%; 9.5% vs. 9.0%	Not reported^f^; Not reported^g^
Wink et al. (2018) [21]	350	USA	Autism Inpatient Collection	ADOS-2	4–21	Not reported	ns^h^
Mandell et al. (2008)^i ^[22]	60,641	USA	Centers for Medicare and Medicaid Services Medicaid Analytic Extract (2001)	ICD-9 code 299.00, 299.8, or 299.9 associated with Medicaid reimbursed claim in 2001	0–21	11% vs. 14%; 3% vs. 4%	Not reported^j^; Not reported^k^
Khanna et al. (2013) [23]	1,330	USA	Medicaid FFS Administrative-Claims	ICD-9 Autism in records	0–65	14.1% vs. 17.4%	ns^l^

Use of other drugs

In total, five clinical studies investigated the use of “other” subsets of medications (Table 7). One study reported only crude statistics. The second study found that females were more commonly prescribed antianxiety (tranquilizers) and non-barbiturates than males. While a third study found that melatonin was prescribed more frequently to females. However, the other two did not observe any statistical difference in the use of metformin, gastrointestinal-related, or sleep-related medications between autistic men and women.

**Table 7:** Use of other drugs male versus female. Significant results are highlighted in bold. ^a^ Other drugs, including piracetam, biperiden, and naltrexone, could not be incorporated into major medication classes. Significance was not reported; ^b ^Antianxiety medications (tranquilizers) per ATC were more commonly prescribed in females (p ≤ 0.02); ^c^ Non-barbiturates per ATC were most widely prescribed in females (p ≤ 0.02); ^d^ Melatonin, relative risk = 1.13, 95% confidence interval = 1.04–1.22 (M < F); ^e^ No significance was associated with sex difference with metformin, sleep aids, and gastrointestinal drugs. Sleep aids included diphenhydramine, clonidine, melatonin, and trazodone. Gastrointestinal drugs were bisacodyl, dicyclomine, docusate, esomeprazole, fiber wafer, lactase, lactulose, lansoprazole, omeprazole, pantoprazole, polyethylene glycol, probiotic, psyllium, ranitidine, senna, and sennosides; ^f^ Other drugs included antimanic drugs (lithium and memantine), anticonvulsants (valproic acid and carbamazepine), and beta-adrenergic blockers. ADI-R: Autism Diagnostic Interview-Revised; ADOS-2: Autism Diagnostic Observation Schedule-Second Edition; ATC: Anatomical Therapeutic Classification; DSM-IV: Diagnostic and Statistical Manual of Mental Disorders-Fourth Edition; DSM-IV-TR: Diagnostic and Statistical Manual of Mental Disorders-Fourth Edition-Text Revision; F: female; FFS: fee-for-service; ICD-9: International Statistical Classification of Diseases and Related Health Problems-Ninth Revision; ICD-10: International Statistical Classification of Diseases and Related Health Problems-Tenth Revision; ICD-10-AM: International Statistical Classification of Diseases and Related Health Problems-Tenth Revision-Australian Modification; M: male; ns, not significant

Author(s) (Year)	Sample size	Location	Means for participant identification (Year)	Diagnosis	Age (Year)	Crude % (M vs. F)	Significance
Memari et al. (2012) [11]	345	Iran	Autism-specific schools in Tehran, Iran (2010–2011)	DSM-IV-TR criteria and ADI-R	7–14	5.4% vs. 13%^a^	Not reported
Satoh et al. (2016) [33]	3,276	Japan	Korean National Health Insurance Claims Database (2009– 2013)	ICD-10, F84.0, F84.1, F84.2, F84.3, F84.4, F84.5, F84.8, F84.9	2–18	Not reported	M < F^b,c^
McLay et al. (2021) [35]	11,202	New Zealand	Integrated Data Infrastructure Administrative Health Data	DSM-IV, ICD-10-AM, or Disability Services Data (Socrates) codes in database	0–18	22.2% vs. 25.4%	M < F^d^
Wink et al. (2018) [21]	350	USA	Autism Inpatient Collection	ADOS-2	4–21	Not reported	ns^e^
Khanna et al. (2013) [23]	1,330	USA	Medicaid FFS Administrative-Claims	ICD-9 Autism in records	0–65	18.4% vs. 23.1%	ns^f^

In autistic patients, a gender-specific difference in psychotropic medication use can be explained by one of the following: (1) the different prescription trends mimic gender-specific trends that also exist in the non-autistic population, or (2) the prescription patterns are due to different comorbidities that autistic males and females experience. In a large cohort (n = 20,194) of autistic patients in the United Kingdom, the three most common neuropsychiatric comorbidities across all patients included behavioral and conduct disorders, anxiety, and ADHD. Behavioral and conduct disorders and ADHD were more common in males, while anxiety and depression were more common in females [17]. It follows suit that prescription patterns would likely differ under these comorbidities. The limitations can partly explain the breadth of data and lack of homogeneity between studies in the present review. Assessing the prevalence of psychotropic medication can be misleading because this analysis limits the visibility into medication trends within specific classes of drugs.

This review highlights the lack of uniformity between studies as the primary limitation. Although several studies overlapped, their inclusion criteria varied (age, IQ, psychiatric comorbidities). Further, medications in some studies were reported from caregivers, while in others, they were directly from electronic medical records. The setting or location also varied, as some studies focused on in-patient and acute care settings. In addition, it was worth considering that geography and health care varied in different countries and regions.

Another drawback is the variability in categorizing drug classes. For example, stimulants were generally thought of as being employed to improve measures of impulsivity and hyperactivity. Still, some stimulants, such as extended-release guanfacine, were also efficacious in a randomized, placebo-controlled trial in reducing oppositional and repetitive behaviors in children with autism and ADHD [36]. Therefore, it must be considered that some stimulant medications might be prescribed to manage symptoms more consistent with mood disorders (such as opposition behavior) than their typical use as attention-related symptoms (hyperactivity).

In general, the lack of efficacious medications for the core symptoms of autism made it challenging to identify trends between sexes. Nonetheless, several studies found substantial differences in prescription patterns and specific classes of drugs. For example, antidepressants, anticonvulsants, and mood stabilizers were more commonly used in females, while stimulants and other ADHD drugs and antipsychotics were more frequently used in males. This review highlights prescription differences between autistic men and women. Such differences in drug use support the idea that autism manifests differently in women than men, so it should not be equated. The evidence presented in this review warrants further investigation to adequately address and compare patterns in psychotropic drug usage by gender, age, IQ, and comorbidity to draw more definitive conclusions in autistic patients.

Another crucial factor, although not within the scope of this review, is the pharmacogenetic testing of autistic males and females to maximize the treatment benefits of psychotropic drugs administered to such patients. The most commonly prescribed psychotropics used in treating autistic patients are metabolized by cytochrome 2D6, an isoenzyme of cytochrome P 450. Depending upon the genetic or chromosomal abnormality, a patient may be a slow or fast metabolizer of psychotropics [37], directly affecting the plasma concentration of these medications and, consequently, their benefits and adverse effects. An improved understanding of the male and female autistic phenotypes and the gender trends of psychotropic prescription based on the genetic profile would help tailor the treatment plan to the specific needs of autistic patients and will lead to better patient outcomes.

## Conclusions

This review highlights the trend of psychotropic drug use in autistic men and women. By reviewing the existing literature and focusing on a symptom-focused model of pharmacologic treatments, we identified a difference in the use of psychotropics among autistic men and women. Autistic women are most likely to consume antidepressants, anticonvulsants, and mood stabilizers. In comparison, autistic men are more likely to use stimulants and ADHD drugs. Moreover, based on the clinical evidence, it is clear that psychiatric comorbidity alone does not entirely explain the differences in these medication usages between genders. Recognizing a female phenotype of autism may partly contribute to this difference but merits further clinical investigation. Understanding the gender trends in psychotropics use by autistic patients is critical as gender-related differences can impact a wide range of pharmacokinetic and pharmacodynamic parameters. These differences may influence the diagnosis and ultimately affect the recommendation for initial dosing and titration of these drugs.
